# Investigation of finishing of leather for inside parts of the shoes with a natural biocide

**DOI:** 10.1038/s41598-020-60285-y

**Published:** 2020-02-26

**Authors:** Elżbieta Bielak, Ewa Marcinkowska, Justyna Syguła-Cholewińska

**Affiliations:** 1 Department of Non-food Product Quality and Safety, Institute of Quality and Product Management Sciences, Cracow University of Economics, Rakowicka 27, Cracow, 31-510 Poland; 2 Department of Microbiology, Institute of Quality and Product Management Sciences, Cracow University of Economics, Rakowicka 27, Cracow, 31-510 Poland

**Keywords:** Environmental economics, Environmental impact, Occupational health, Risk factors

## Abstract

The prevention of decrease of quality caused by microbial activity in footwear materials entails the use of biocides. However, these substances may pose a hazard to humans and to the natural environment. The paper presents the results of antimicrobial effect investigation for cowhide leather treated with oregano oil. In these studies oil was applied by spraying onto the finished leather surface and examined to determine its antimicrobial activity by using the Agar Diffusion Plate Test. These results were compared with those where a cowhide leather was treated with oil at the stage of fatliquoring. In addition, the oregano oil toxicity level was assessed and compared with biocides used in the tanning industry. Introducing oregano oil into the leather at the stage of fatliquoring provides a better antimicrobial effect than by spraying, however hygienic finishing of leather can be obtained by introducing oil into the raw material by these both methods. The oregano oil is characterised by the lowest number of hazards and toxicity as compared with commercial biocides. The use of essential oils as natural biocides in the tanning industry seems to be especially important and suitable solution considering the harmful effects of synthetic biocides to humans and the environment.

## Introduction

Due to its characteristic properties, animal skin is a valuable raw material used in the footwear industry. The properties include absorption and water vapour permeability and sweat absorbability, i.e. properties of utmost importance for leather used in the manufacture of inside parts of the shoe. Due to the properties mentioned above and many others, natural leather is gaining an advantage over their substitutes, namely leather-like materials, especially with regard to hygienic properties.

In particular, hygienic properties of footwear should be analysed for the presence and kinds of microorganisms colonizing the inside of used shoes. Favourable conditions created by moisture and temperature inside the shoe as well as lack of attention to its cleanliness and improper maintenance may lead to the growth of bacteria and fungi, including those pathogenic and responsible for skin infections and re-infections. Szostak-Kot^1^ indicates that footwear use leads to its contamination with such bacteria as *Staphylococcus aureus* or *Bacillus megaterium*. However, athlete’s foot (tinea pedis) is caused by such species as: *Epidermophyton floccosum*, *Trichophyton mentagrophytes*, *Trichophyton verrucosum*, *Microsporum gypseum* and some yeasts belonging to the genus *Candida*.

The prevention of microbial growth and multiplication in footwear materials and shoes entails the use of biocides, i.e. bactericidal or biostatic chemicals. According to the Regulation (EU) No 528/2012 of the European Parliament and of the Council^2^ biocidal products are used to control organisms that are harmful to human or animal health, and may cause damage both to industrial products, man-made materials or those of natural origin. In addition, due to their specific features and uses, biocidal products may pose a hazard to humans and animals as well as to the natural environment. According to Annex V to the said document, biocides used in the tanning industry for leather preservation are intended to prevent harmful effects of microbes by preventing the settlement of microorganisms on the surface of materials, thus hampering the development of unpleasant odours, and to gain other benefits from the activity of biocides^2^.

It follows from the definition of biocidal products given above that they may pose a hazard to humans and their surrounding environment because of their properties. For some biocides used in the tanning industry their harmfulness was confirmed and these agents were withdrawn from use or subjected to usage restrictions. An example is dimethyl fumarate (DMF) that, as the research suggests, penetrates the skin causing local acute contact urticaria and symptoms such as pain, irritation, burns, reddening and swelling^3–5^. Information about DMF could be found in REACH Regulation^6^, i. e. Regulation of the European Parliament and of the Council concerning the Registration, Evaluation, Authorisation and Restriction of Chemicals. This regulation was introduced to improve the protection of environment and human health against the risk which is posed by the chemical substances. It lays down collecting procedures and assessing information on the properties of substances and the hazards associated with them. According to REACH^6^ for DMF the restriction imposed limits the possible content of this substance in a product or even in its parts to a concentration not exceeding 0.1 mg/kg. Another example is pentachlorophenol (PCP), an agent hazardous to human health and life^7^ which was used to protect hides and skins in the pickling process; it has, however, been withdrawn from use for environmental reasons^8^. The REACH^6^ regulates that PCP as a substance or even as a constituent in other substances/mixtures must not be present in a concentration equal to or greater than 0.1% by mass. PCP was the most commonly used fungicide in the tanning industry until the 1990s. It has been superseded by 2-(thiocyanomethylthio)benzothiazole (TCMTB)^9^. Unfortunately, according to previous studies^10^, DMF, PCP and other chemicals such as chromium (VI) and formaldehyde are still present in too large amounts in leather products sold in the world market, as confirmed by The Rapid Alert System for Dangerous Non-food Products Data^11^. The fact that the presence of harmful chemicals in products and tanning wastewater is a topical and important issue is supported by numerous studies carried out for many years^12–14^ and aimed at making the tanning industry more ecological and environmentally friendly.

One of the important classes of human health hazards is acute toxicity that according to the CLP Regulation^15^ (Regulation of the European Parliament and of the Council on classification, labelling and packaging of substances and mixtures which is based on the so-called globally harmonized system of classification and labelling of chemicals (GHS), elaborated by United Nations, which was prepared to detect the hazardous chemicals and inform users about the risks associated with them) means adverse effects of oral or dermal administration of one dose of a substance or mixture, or multiple doses given within a day, or an inhalation exposure of 4 hours. The classification system of substances acute toxicity, allow to assign the substance to the one of four toxicity categories, based on the quantitative criteria^15^. Stephenson *et al*.^16^ carried out the investigations of acute toxicity of pure pentachlorophenol and technical formulation of PCP to three species of *Daphnia*, taking into account the influence of various factors, i.e.: the time of exposition, the age of tested organism, and pH of investigated solution. They proved that PCP caused the toxic reaction in a narrow range of concentrations, and the reaction is the strongest just after the exposition (0 to 24 hours). Depending on the species, LC50 (lethal concentration 50%) estimated for adult daphnids were in the range 0.51–4.59 mg/L for pure PCP and 0.33–3.66 mg/L for PCP which had technical level of purity. St. Omer and Gadusek^17^ proved the LD50 (lethal dose 50%) of PCP, which had technical level of purity, depends on the age of rats. For the suckling rats (age 10 and 20 days) the LD50 was 50–180 mg/kg, for young rats (25 to 50 days) it was 220–230 mg/kg, whereas for adult rats (70 and 134 days) it was 80–120 mg/kg. The investigations carried out in other experiment with rats allow to prove that the LD50 of DMF depends on the gender of rat (males – 3220 mg/kg, females 2630 mg/kg)^18^. Colosio *et al*.^19^ described in their article the toxicological and immune health effects of more than 30 people exposed to prolonged exposure to PCP. The results of these investigations proved also that the PCP of technical level of purity had an influence on the functional immune response. Also the investigations of Daniel *et al*.^20^ carried out for 190 patients, which have been exposed to pesticides containing PCP for more than 6 months proved that, PCP had weakened the cellular and humoral immunity. The investigations of biocides toxic effects are also described in many others articles^21–24^.

In consideration of the harmful effects of biocides investigation was undertaken into the option of replacing these agents with natural and environmentally friendly substances of vegetable origin, i.e. essential oils with antiseptic properties. Scientists such as Bayramoĝlu^25^, Bayramoĝlu *et al*.^26^ and Širvaitytė *et al*.^27^ applied oils at different stages of processing animal hides for leather (wetting, pickling, tanning, fatliquoring) at various concentrations when considering protection of raw materials against damage caused by the presence of microorganisms. The tests, among other things, included a comparison of the effects of essential oils with those of commercially available biocides used in tanning, and it has been found that natural substances (at appropriate concentrations) were better at controlling microorganisms and protecting raw materials. Kaygusuz and Yaşa^28^ also paid attention to deterioration of hide resulting from microbial activity and to preventing it by using natural bioactive materials, such as essential oils or vegetable extracts. A solution in which skins are protected against harmful effects of microorganisms by using a plant material has been proposed by Mohammed *et al*.^29^. In this study they used dried and powdered roots of *Rumex abyssinicus* (mekmeko) to reduce the use of common salt to preserve raw goat skins and found that this was a cleaner alternative to the conventional salt-based preservation method. Information about studies performed by various researchers in 2006–2015 on the use of plant-based preservatives in the leather industry was gathered and presented also by Wu *et al*.^30^.

In previous studies^31–33^ the authors made attempts to introduce various essential oils at different concentrations into lining leather to find the oil and its concentration that could ensure the best and long-lasting antimicrobial effect of leather. Microbiological studies confirmed that the antimicrobial effect persisted even after 12 months for leather fatliquored with added oregano oil at a concentration of 3% by leather mass. This enables to conclude that hygienic properties of lining leather as well as footwear components made of leather treated in such a manner can be significantly improved by introducing oregano oil^33,34^.

The purpose of the investigation was to determine antimicrobial activity of sock lining leather finished using the method of spraying with oregano oil with antimicrobial action which is an alternative to synthetic biocides. A comparison was made of the effectiveness and durability of the finishing with the effect that was achieved by introducing the oil into the lining leather structure at the stage of fatliquoring.

## Materials and Methods of Microbiological Tests

### Materials

#### Leather

For the purpose of this study, cowhide finished leather intended for sock lining was used. The leather was treated by oil spraying. In order to perform a comparative analysis, the results obtained in previous investigation of wet-blue cowhide lining leather, prepared without using synthetic biocides in tannery industry process, was used. This leather was treated with oregano essential oil at the stage of fatliquoring in a laboratory^32–34^.

#### Essential oil

The oregano oil used in the testing was acquired from the Laboratory of Industrial and Experimental Biology at the State Higher Vocational School in Krosno (Poland). The oil was produced in Portugal by distillation with water vapour, as declared by the manufacturer, from plant *Origanum vulgare*. Verification of the composition of the oil was done by gas chromatography-mass spectrometry^33^ and showed that the main component was carvacrol (94.06% of the composition); trace amounts of other components were also detected, i.e. palmitic acid ethyl ester (0.15% of the composition), indene derivative (0.07% of the composition), hexadecanoic acid (0.14% of the composition), erucic acid (0.14%). 5.44% of the composition were other compound which could not be identified in the chromatograph.

#### Microorganisms and media

The tests examined the resistance of leather treated with oregano oil to the following microbial strains:

- *Staphylococcus aureus* (ATCC 25923) (Gram-positive);
- *Escherichia coli* (ATCC 25922) (Gram-negative);
- *Pseudomonas aeruginosa* (ATCC 27853) (Gram-negative);
- *Candida albicans* (ATCC 10231).

Two substrates were used in the tests:

- agar TSA (Trypticase soy agar) – bacterial culture medium;
- Sabouraud dextrose agar with the addition of glucose (4%) – fungal culture medium.

### Antimicrobial leather treatment

#### Wet-blue leather treatment with oregano oil at the stage of fatliquoring

In the first method, wet-blue cowhide lining leather pieces were treated by introducing oregano oil at the selected concentration of 3% by leather mass during the fatliquoring process. This concentration was selected from three concentrations that were tested, i.e. 1%, 3% and 5%, as the optimal one, i.e. such that enables obtaining good and lasting antimicrobial effect^32–34^. The post-tanning processes in wet-end with an addition of oregano oil are presented in Table 1.

**Table 1:** The post-tanning processes in wet-end^33^.

	Process stage						
	I soaking	II retanning	III rinsing	IV retanning	V fatliquoring	VI fixation	VII rinsing
Chemical compound/agent used and its percentage to shaved leather mass %	water – 250	water – 150 chromitan B – 3 baking soda – 0.2 sodium formate – 2 baking soda – 1.7	water – 250	water – 100 perfectol HQ – 1 relugan RE – 3 mimose – 5 relugan RE – 2 water – 50	water – 100 ammonia water – 0.2 perfectol HQ – 5 dekalin SE – 0.2 **oregano oil – 3**	water – 100 formic acid – 0.5–1.6	water – 200
Process temperature °C	35	35	35	40	45	45	45
Bath pH (at the end of stage)	—	5.5–5.8	—	—	—	3.4–3.6	—

Antimicrobial activity was determined after 1, 6 and 12 months of leather finishing. The bacterial and fungal strains potentially pathogenic for humans and capable of causing infections and diseases of the foot skin, i.e. *Staphylococcus aureus, Staphylococcus epidermidis, Escherichia coli, Candida albicans, Scopulariopsis brevicaulis* were used^32–34^. The control samples were specimens of wet-blue leather that were fatliquored without the addition of oil.

#### Treatment of sock lining leather finished with oregano oil by surface spraying

For the microbiological tests, round-shaped laboratory samples with a diameter of 25 ± 5 mm were collected from finished cowhide leather intended for sock lining by means of a press and appropriate punching tool. The samples were sprayed manually by means of an atomiser, directly without solving, with oregano oil from approx. 8–10 cm; the oil was applied in duplicate (each time the volume applied was 1 mL). The non-mechanical method of applying the oil was intended to imitate the actual conditions of use when the preparation is applied by the consumer on their own. The samples were left to dry in horizontal position at room temperature (approx. 22–25 °C). After the drying it was observed that the original beige colour of the leather became yellowish after having been treated with oregano oil.

After 5 days some of the samples were subjected to microbiological testing while the other samples were further stored in paper envelopes and then subjected to testing after 30 days of the time of spraying. When oil is applied by means of spraying, leather samples do not require such a long storing time in order to remove excess moisture as it was the case when the oil was introduced during the fatliquoring process. Therefore, the tests could be performed already after 5 days of the oil application. The leather specimens not sprayed with the oil, i.e. the control samples, were stored separately under the same conditions.

### Microbiological examination of leather treated by surface spraying

Microbiological examination was carried out according to the ISO 20645 standard^35^. The same method was used in previous experiments where antimicrobial activity was examined for leather to which oregano oil was introduced at the stage of fatliquoring at a concentration of 3% by leather mass^32–34^.

Before testing the leather specimens (disks of 25 ± 5 mm) were conditioned at room temperature, 22–25 °C for 24 hours in sterile Petri dishes. Reference strain suspensions were prepared from 24-hour bacterial cultures in sterile 0.9% NaCl solution and brought to a 0.5 McFarland standard (1.5 × 10^8^ cfu/mL) based on densitometric measurements with DENSIMAT densitometer (bioMerieux). Yeast suspensions were prepared from cultures of *Candida albicans* and adjusted to the optical density of the McFarland standard No. 1 (3.0 × 10^8^ cfu/mL). The Petri dishes containing agar were inoculated with 0.1 mL of these microbial suspensions. The disks of leather facing the grain and flesh sides up were placed centrally in the Petri dishes using sterile tweezers. The experiment was carried out in duplicate.

Microbes were cultured in laboratory incubator for:

- 18 to 24 hours for bacteria at 37 ± 1 °C;
- 48 hours for yeasts at 37 ± 1 °C.

After incubation the presence of a zone of inhibition of microbes around the specimen as well the presence or lack of microbial growth on agar under the specimen were evaluated. Simultaneously, the control specimen (leather not sprayed with oil) was tested by using the procedure described above. The effect of antimicrobial activity of cowhide leather for sock lining treated by surface spraying was assessed with respect to reference strains of bacteria and yeasts according to the scale shown in ISO 20645 standard^35^ (see Table 2).

**Table 2:** Antibacterial effect of the antibacterial treatment^35^.

Inhibition zone (mm) Mean value	Growth^a/^	Description	Assessment
>1	none	inhibition zone exceeding 1 mm, no growth^b/^	good effect
1–0		inhibition zone up to 1 mm, no growth^b/^	
0		no inhibition zone, no growth^c/^	
0	slight	no inhibition zone, only some restricted colonies, growth nearly totally suppressed^d/^	limit of efficacy
0	moderate	no inhibition zone, compare to the control growth reduced by half^e/^	insufficient effect
0	heavy	no inhibition zone, compared to the control no growth reduction or only slightly reduced growth	

^a/^The growth of bacteria in the nutrient medium under the specimen.

^b/^The extent of the inhibition shall only partly be taken into account. A large inhibition zone may indicate certain reserves of active substances or a weak fixation of a product on the substrate.

^c/^The absence of bacterial growth, even without inhibition zone, may be regarded as a good effect, as the formation of such inhibition zone may have been prevented by a low diffusibility of the active substance.

^d/^As good as no growth indicates the limits of efficacy.

^e/^Reduced density of bacterial growth means either the number of colonies or the colony diameter.

## Results and Discussion of Microbiological Tests

### Antimicrobial effect of leather treated at the stage of fatliquoring

When assessing antimicrobial effect of leather fatliquored with oregano oil (3%) after one month, a very good antimicrobial activity of the leather was confirmed by considerable zones of growth inhibition for mould *Scopulariopsis brevicaulis* (>32 mm), yeasts *Candida albicans* (16 mm), bacteria *Staphylococcus aureus* and *Staphylococcus epidermidis* (10–11 mm) and the strain of *Escherichia coli* (9 mm)^34^. After 6 months of fatliquoring, a good antimicrobial effect against all the microorganisms in the test was still observed. The sizes of the zones of growth inhibition varied between 8–10 mm for *Staphylococcus epidermidis* up to>32 mm for *Scopulariopsis brevicualis*^32^.

In an experiment performed after 12 months of storage of the samples, the antifungal activity of leather with oregano oil (3%) was reduced with regard to filamentous fungi *Scopulariopsis brevicaulis*. In this case, the growth inhibition zone was equal to 0. For the other microorganisms, zones of considerable sizes were still observed (in the range between 4 mm and 15–17 mm), which demonstrated the strong antimicrobial effect^34^. For the control samples prepared without using the essential oil, no grow inhibition zones were observed around the leather disks either after 1, 6 or 12 months of storage. Under the samples, zero, slight or moderate growth of the reference strains was observed^32,34^.

The results presented allowed to conclude that lining leather fatliquored with the addition of oregano oil at a concentration of 3% by leather mass is characterised by good and durable antimicrobial effect persisting even for 12 months. The persistence of biostatic properties of the leather samples for such a long time resulted from the fact that the decrease in emission of carvacrol, i.e. the active component of the oil, was almost two times lower in comparison with the decreases in emissions of all the volatile organic compounds during the storage of the samples. This was corroborated in earlier experiments using the method of gas chromatography with mass spectrometry^33^.

### Antimicrobial effect of leather treated by surface spraying

**Figure 1:**
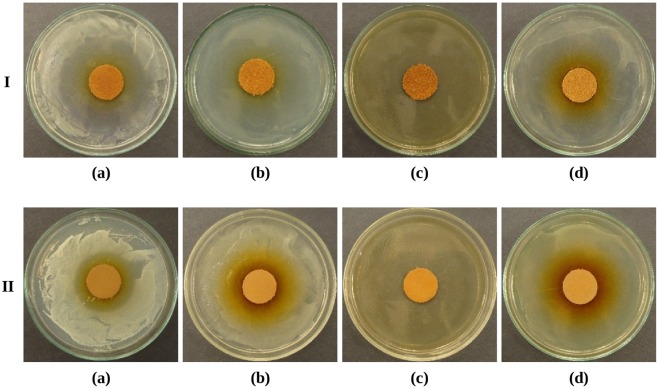
The leather sprayed with oregano oil on the grain side (I row) and flesh side (II row), after 5 days of storage, growth inhibition zones of *Staphylococcus aureus* (**a**), *Escherichia coli* (**b**), *Candida albicans* (**c**), *Pseudomonas aeruginosa* (**d**).

The results of antimicrobial activity tests for the leather sprayed with oregano oil are presented in Table 3. At 5 days after spraying considerable inhibition zones of microbial growth were observed both around the leather specimens sprayed on the grain side and on the flesh side. For specimens treated on the grain side the inhibition zones of the following size were noted: *Staphylococcus aureus* −18 mm (Fig. 1Ia), for *Escherichia coli* −25 mm (Fig. 1Ib), *Candida albicans* >32 mm (Fig. 1Ic). Weaker, but noticeable effect of leather microbial activity against *Pseudomonas aeruginosa*, which are resistant to antimicrobial agents, was also recorded in the form of a 1 mm inhibition zone (Fig. 1Id). For specimens sprayed with oil on the flesh side, the antimicrobial effect achieved was slightly weaker for *Staphylococcus aureus* (8–11 mm) (Fig. 1IIa) and *Escherichia coli* (6 mm) (Fig. 1IIb), and for *Pseudomonas aeruginosa* (Fig. 1IId), where no inhibition zone was observed. Equally strong (as on the grain side) antimicrobial effect was observed for yeast *Candida albicans*, where the inhibition zone was >32 mm (Fig. 1IIc). No microbial growth under the specimens was observed.

**Table 3:** An assessment of antimicrobial effect of leathers surface sprayed with oregano oil 5 and 30 days after spraying.

Microorganisms	Growth inhibition zones around specimens [mm], microorganism growth evaluation according to the scale^35^			
	Leather sprayed with oregano oil			
	After 5 days		After 30 days	
	Grain side	Flesh side	Grain side	Flesh side
*Staphylococcus aureus*	18	8–11	6–10	1–3
	>1 no growth under the specimen	>1 no growth under the specimen	>1 no growth under the specimen	>1 no growth under the specimen
*Escherichia coli*	25	6	2–6	0–1
	>1 no growth under the specimen	>1 no growth under the specimen	>1 no growth under the specimen	>1 no growth under the specimen
*Pseudomonas aeruginosa*	1	0	0	0
	>1 no growth under the specimen	0 no growth under the specimen	0 no growth under the specimen	0 slight growth under the specimen
*Candida albicans*	>32	>32	11–13	15–18
	>1 no growth under the specimen	>1 no growth under the specimen	>1 no growth under the specimen	>1 no growth under the specimen

**Figure 2:**
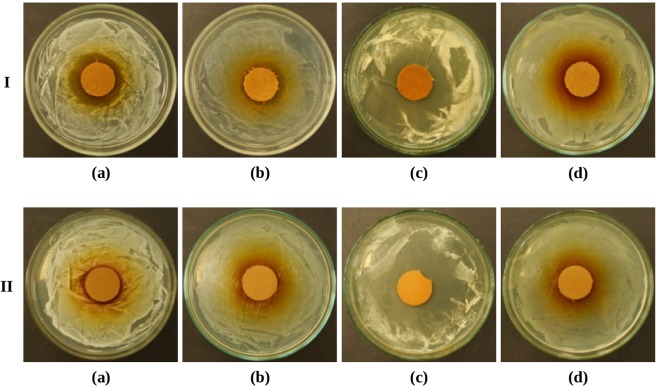
The leather sprayed with oregano oil on the grain side (I row) and flesh side (II row), after 30 days of storage, growth inhibition zones of *Staphylococcus aureus* (**a**), *Escherichia coli* (**b**), *Candida albicans* (**c**), *Pseudomonas aeruginosa* (**d**).

After 30 days the antimicrobial effect of the leather was attenuated (Table 3). For samples sprayed with oil on the grain side, the growth inhibition zones observed were of the following sizes: for *Staphylococcus aureus* – 6–10 mm (Fig. 2Ia); for *Escherichia coli* – 2–6 mm (Fig. 2Ib); for *Candida albicans* – 11–13 mm (Fig. 2Ic). For the bacteria *Pseudomonas aeruginosa*, the growth inhibition zone was reduced to 0 mm (Fig. 2Id).

When analysing the results obtained for samples sprayed on the flesh side, a reduction in the antimicrobial activity of the leather was also observed. The largest growth inhibition zone was observed for the yeast *Candida albicans* (15–18 mm) (Fig. 2IIc). For the bacteria *Staphylococcus aureus*, a zone with a diameter of 1–3 mm (Fig. 2IIa) was observed; and for *Escherichia coli* it was 0–1 mm (Fig. 2IIb). The antimicrobial effect of leather on the Gram-negative bacterium *Pseudomonas aeruginosa* was attenuated to such an extent that slight bacterial growth occurred under the sample (Fig. 2IId), which was not observed for any other sample tested after 30 days of spraying.

**Figure 3:**
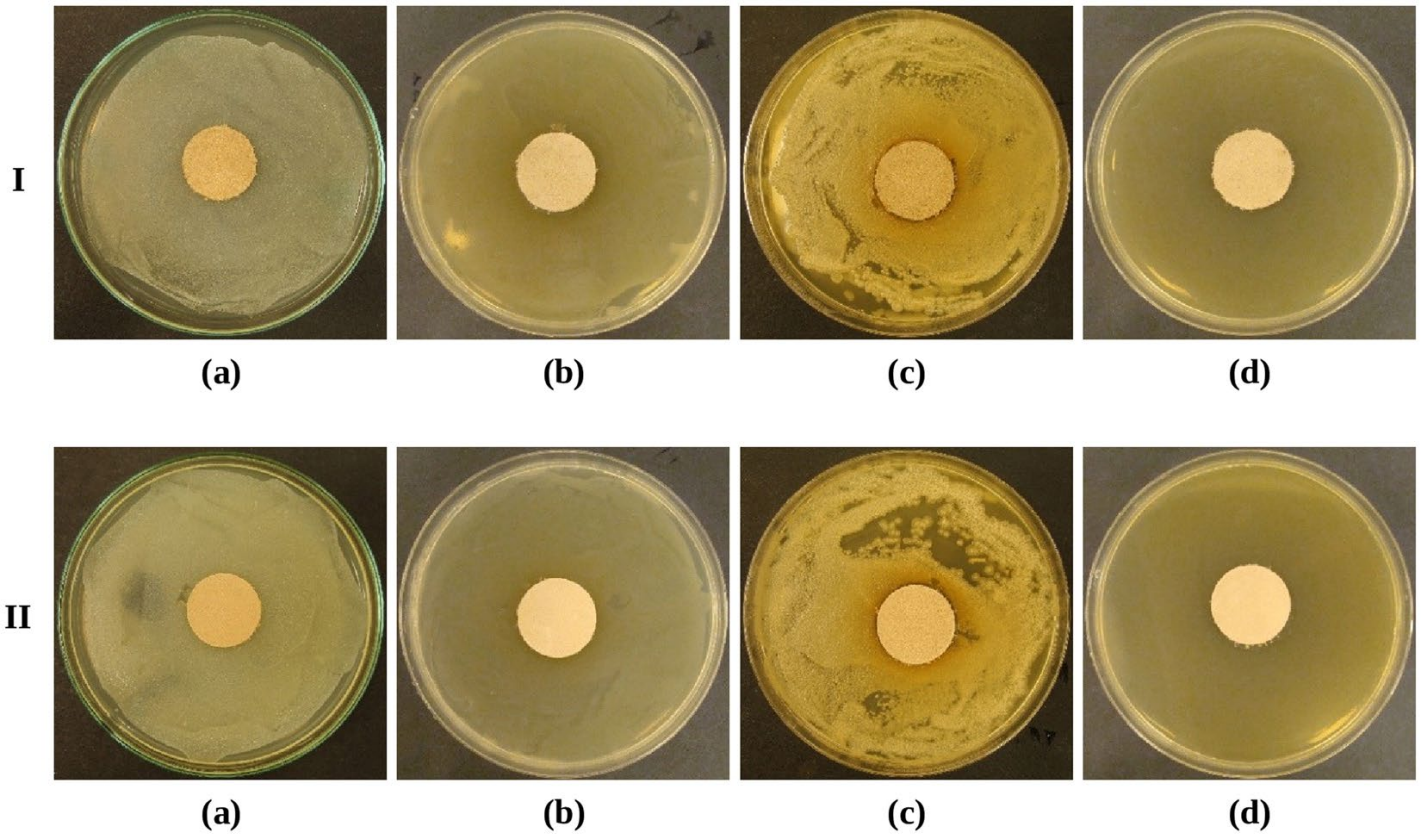
The leather not sprayed with oregano oil (control group), the grain side (I row) and flesh side (II row), no growth inhibition zones of *Staphylococcus aureus* (**a**), *Escherichia coli* (**b**), *Candida albicans* (**c**), *Pseudomonas aeruginosa* (**d**).

In the tests performed for the control group of samples (Table 4) no microbial activity was observed. No typical growth inhibition zones occurred around the samples (Fig. 3) and zero, slight or moderate microbial growth occurred under the samples.

**Table 4:** An assessment of antimicrobial effect of leathers not sprayed with oregano oil (control group).

Microorganisms	Growth inhibition zones around specimens [mm], microorganism growth evaluation according to the scale^35^	
	Leather without oregano oil (control group)	
	Grain side	Flesh side
*Staphylococcus aureus*	0	0
	0 slight growth under the specimen	0 slight growth under the specimen
*Escherichia coli*	0	0
	0 no growth under the specimen^a/^	0 no growth under the specimen^a/^
*Pseudomonas aeruginosa*	0	0
	0 no growth under the specimen^a/^	0 no growth under the specimen^a/^
*Candida albicans*	0	0
	0 no growth under the specimen^a/^	0 moderate growth under the specimen

^a/^Strong specimen adhesion; should not be interpreted as a good effect.

When comparing antimicrobial activity of leather treated using the spraying method with the activity of leather fatliquored with oregano oil after 30 days of storage, it should be noted that it was possible to achieve a good effect of antimicrobial treatment by both methods. Although the samples refined by fatliquoring had stronger effect, microbial growth inhibition zones of considerable sizes were also observed for samples treated by the spraying method. The test results show that surface spraying with oregano oil is one of the possible methods that can be applied in order to refine sock lining leather and give it antimicrobial properties. Although the effect is attenuated over time, the experiment indicates that this effect is fully sufficient to protect the inside of the shoe against multiplication of microorganisms hazardous to human health for some time. These microorganisms may also contribute to decomposition of footwear materials. Reapplication of the oil finish by the user (e.g. by spraying with an atomizer) would allow maintaining antiseptic properties at the desired level for a longer time, thus eliminating the need to use chemical antimicrobial agents, e.g. foot and shoe deodorant sprays as well as biocides in raw material finishing. What is more, the proposed method of direct spraying can be used for shoe insoles, also those made of materials other than leather, e.g. textiles or leather-like materials. These shoe components treated with oregano oil and replaced by the user at appropriate intervals might enhance hygienic properties of shoes understood in the context of antimicrobial activity.

### Estimation of the toxicity level of oregano oil and synthetic biocidal preparations used in the tanning industry

The results of toxicity assessment for oregano oil and commercial biocidal preparations used in tanning and marketed under specified trade names are presented in Table 5. These results are based on biocidal material safety data sheets (MSDS), containing the data that allow the acute toxicity estimate (ATE) values to be derived, and information on acute toxicity of these biocidal substances. This assessment included the four main areas of toxicity effects of dangerous substances including hazards resulting from oral, inhalation and dermal route of exposure as well as eye contact.

**Table 5:** The estimation of the toxicity level of oregano oil and selected commercial biocidal preparations used in the tanning industry.

Health hazard type (with H-codes^a/^)	Category			
	1	2	3	4
**Oregano essential oil – carvacrol (5-isopropyl-2-methylphenol) 94.06%**				
harmful if swallowed (H302)				✓
causes skin irritation (H315)		✓		
causes serious eye damage (H318)	✓			
effects on respiratory system	no data available			
**Biocide 1 – a mixture containing 2-(thiocyanomethylthio)-benzothiazole and 2-methylpropan-1-ol**				
harmful if swallowed (H302)				✓
causes skin irritation (H315), may cause an allergic skin reaction (H317), causes severe skin burns and eye damage (H314)	✓			
causes serious eye irritation (H319), causes severe skin burns and eye damage (H314), cause serious eye damage (H318)	✓			
fatal if inhaled (H330), may cause respiratory irritation (H335), may cause drowsiness or dizziness (H336)	✓			
**Biocide 2 – a mixture containing 2-(thiocyanomethylthio)-benzothiazole and diethylene glycol ethyl ether**				
harmful if swallowed (H302)				✓
causes skin irritation (H315), may cause an allergic skin reaction (H317)	✓			
causes serious eye irritation (H319)		✓		
fatal if inhaled (H330)	✓			
**Biocide 3 – a mixture containing chlorocresol sodium and sodium 2-phenylphenoxide**				
harmful if swallowed (H302)				✓
harmful in contact with skin (H312), causes skin irritation (H315), causes severe skin burns and eye damage (H314)	✓			
causes serious eye irritation (H319), causes severe skin burns and eye damage (H314), cause serious eye damage (H318)	✓			
may cause respiratory irritation (H335)			✓	
**Biocide 4 – a mixture containing 4-chloro-3-methylphenol, 2-octyl-2H-isothiazol-3-one and biphenyl-2-ol**				
harmful if swallowed (H302)				✓
toxic in contact with skin (H311), harmful in contact with skin (H312), causes skin irritation (H315), may cause an allergic skin reaction (H317), causes severe skin burns and eye damage (H314)	✓			
causes serious eye irritation (H319), causes severe skin burns and eye damage (H314), cause serious eye damage (H318)	✓			
toxic if inhaled (H331), may cause respiratory irritation (H335)			✓	

^a/^Hazard Statement Codes for Physical Hazards.

The use of substitutes for chemical substances (biocides) to meet the requirements of the Best Available Techniques for the tanning industry requires raw materials of lower toxicity and lower environmental impact to be used at the lowest possible concentration levels^36^. In industrial practice, special care should be taken when working with chemical compounds that pose the greatest hazard and are assigned to category 1 within any hazard group (or such contact should possibly be avoided).

With regard to the number of hazards and the categories of their toxicity Biocide 1 should be considered the most hazardous among the biocidal preparations used in the tanning industry, presented in Table 5. This is a dangerous product containing highly toxic substances and therefore its toxicity has been classified as category 1 in three areas. Among them one deserves special attention: 2-(thiocyanomethylthio)-benzothiazole (TCMTB), it is present in biocide 1 (the range of concentrations 30 to 50%), and also in biocide 2 (the range of concentrations 25 to 30%). The toxicity of this compound and the products of its degradation was assess by Nawrocki *et al*.^37^ in the acute test (48-hours), and the chronic test (7-days), carried out for model species *Ceriodaphnia dubia*. The EC50 (median effective concentration) value measured by authors for TCMTB were 15.3 µg/L and 9.64 µg/L, in the acute and in the chronic tests, respectively. Moreover Nawrocki *et al*.^37^ established that TCMTB was an order of magnitude more toxic than the products of its degradation. The value of LD defined for this compound for other tested organisms can differ. For example LD50 measured for rats directly fed (oral) with TCMTB ranges between 2538 mg/kg or even 679 mg/kg, depending on the data source^38^. The website of PubChem^39^ contains similar data set for acute effects of 4-chloro-3-methylphenol, which is present in biocide 4 (Table 5) at the range of concentrations 30 to 40%. The LD50 value after oral administration to rats was 1830 mg/kg, whereas to mice 600 mg/kg. Biocide 4 contained also 2-octyl-2H-isothiazol-3-one, its concentrations was 8 to 10%. LD50 value of this compound measured for rats (oral administration) was 550 mg/kg^40^.

Oregano oil containing 94% of carvacrol is the most environmentally friendly substance among all the compounds (Table 5) analysed. This oil ingredient known by the full chemical name 5-isopropyl-2-methylphenol has the lowest number of hazards, and in one area only, i.e. eye contact, so it was classified in toxicity category 1. According to research^41^, LD50 value of carvacrol which was orally administrated to rats was 810 mg/kg. Based on the data present in MSDS of carvacrol it can be concluded that none of the ingredients present in concentrations bigger than 0.1% was defined by International Agency for Research on Cancer (IARC) as probable, possible or confirmed carcinogen for humans. In addition, this substance does not contain components considered either persistent, bioaccumulative and toxic, or very persistent and very bioaccumulative (vPvB) at the level of concentration 0.1% or above.

## Conclusions

The results of the investigation indicate that the method proposed in the paper of finishing leather by spraying with oregano oil makes it possible to give it antimicrobial properties. The effect obtained is satisfactory, especially in short term after the application of the substance. Over time, the antimicrobial activity of the leather is attenuated but only to a level that can still be termed as good. The is evidenced by the growth inhibition zones observed after 30 days of the spraying around leather samples, reaching diameters up to 10 mm for *Staphylococcus aureus* and even 18 mm for *Candida albicans*. The results corroborate the possibility to utilise the oil in order to refine leather, which was also demonstrated in previous studies in which oregano oil was introduced into leather during the fatliquoring process. The latter of the proposed methods of application enabled obtaining a stronger and longer lasting antimicrobial effect but it can only be used in the leather production process and not by the consumer according to their individual needs.

The described methods of finishing leather for inside parts of the shoe using oregano oil are innovative as well as human and environment friendly solutions, which will allow to improve the quality of products, taking into account the ecological aspect. The results obtained clearly indicate that hygienic properties of leather and footwear containing inside components made of materials treated in such a way can be significantly improved not only by introducing oregano oil into the raw material at the stage of fatliquoring but also by spraying. The use of this oil provides lasting antimicrobial properties to leather against bacteria (*Staphylococcus aureus, Escherichia coli*) and fungi (*Candida albicans*) capable of causing bacterial and fungal infections, which is particularly important to people who have lowered immunity or suffer from fungal skin diseases, such as fungal foot and nail infections.

The applicability of the proposed methods in the aspect of industrial use is supported by the results of this present analysis and toxicity estimation for oregano oil and synthetic biocides. For carvacrol, the predominant ingredient (94%) in oregano oil, the lowest number of hazards and the lowest toxicity levels were found as compared with commercial biocide preparations. The elimination of synthetic biocides and substituting them with natural ones (e.g. oregano oil) may significantly reduce the emission of toxic compounds from hide processing as well as highly improve workplace safety. Thus, the use of essential oils in the tanning industry as natural biocidal substances seems to be an especially important and suitable solution.

## Data Availability

The datasets generated and analyzed during the current study are available from the corresponding author (Elżbieta Bielak) on reasonable request.
